# Association of Ambient Air Pollution with Respiratory Hospitalization in a Government-Designated “Area of Concern”: The Case of Windsor, Ontario

**DOI:** 10.1289/ehp.7300

**Published:** 2004-12-14

**Authors:** Isaac N. Luginaah, Karen Y. Fung, Kevin M. Gorey, Greg Webster, Chris Wills

**Affiliations:** ^1^Department of Geography, University of Western Ontario, London, Ontario, Canada; ^2^Department of Mathematics and Statistics, and; ^3^School of Social Work, University of Windsor, Windsor, Ontario, Canada; ^4^Canadian Institute for Health Information, Toronto, Ontario, Canada

**Keywords:** air pollution, area of concern, Ontario, respiratory disease, Windsor

## Abstract

This study is part of a larger research program to examine the relationship between ambient air quality and health in Windsor, Ontario, Canada. We assessed the association between air pollution and daily respiratory hospitalization for different age and sex groups from 1995 to 2000. The pollutants included were nitrogen dioxide, sulfur dioxide, carbon monoxide, ozone, particulate matter ≤10 μm in diameter (PM_10_), coefficient of haze (COH), and total reduced sulfur (TRS). We calculated relative risk (RR) estimates using both time-series and case-crossover methods after controlling for appropriate confounders (temperature, humidity, and change in barometric pressure). The results of both analyses were consistent. We found associations between NO_2_, SO_2_, CO, COH, or PM_10_ and daily hospital admission of respiratory diseases especially among females. For females 0–14 years of age, there was 1-day delayed effect of NO_2_ (RR = 1.19, case-crossover method), a current-day SO_2_ (RR = 1.11, time series), and current-day and 1- and 2-day delayed effects for CO by case crossover (RR = 1.15, 1.19, 1.22, respectively). Time-series analysis showed that 1-day delayed effect of PM_10_ on respiratory admissions of adult males (15–64 years of age), with an RR of 1.18. COH had significant effects on female respiratory hospitalization, especially for 2-day delayed effects on adult females, with RRs of 1.15 and 1.29 using time-series and case-crossover analysis, respectively. There were no significant associations between O_3_ and TRS with respiratory admissions. These findings provide policy makers with current risks estimates of respiratory hospitalization as a result of poor ambient air quality in a government designated “area of concern.”

Poor environmental quality has been an important public health issue for some time now. Research using large-scale data sets has shown a fairly consistent relationship between air pollutant levels and respiratory diseases in a variety of communities in the industrialized world (e.g., Atkinson et al. 1999; Dockery et al. 1993; Lin et al. 2002, 2004; Pope et al. 1995; Schwartz 1994).

In Canada, several reports have been published linking air pollution to adverse population health in cities based on data that were collected in the 1980s and early 1990s (e.g., Burnett et al. 1994, 1999; Goldberg et al. 2001). Windsor, Ontario, with a population of 208,402, is one of the cities that has been identified as heavily polluted (Burnett et al. 1998). The city is one of the most industrialized cities in Canada, with major industries including three automobile assembly plants, an engine plant, a foundry, and a scrap metal recycling plant. In addition, there is the outstanding problem of transboundary air and water pollution from the U.S. states of Ohio, Illinois, and Michigan. The city is immediately downwind of major steel mills with associated coking operations in Detroit, Michigan, the wastewater treatment plant of Detroit and associated sludge incineration facilities, and a major power plant that until recently was coal fired. Consequently, Windsor and surrounding communities have been identified as an “area of concern” and in need of further health investigation (Health Canada 2000).

Furthermore, in line with Windsor’s ranking as a city with a high level of pollution compared with other Canadian cities (Burnett et al. 1998), a recent community-health profile by Gilbertson and Brophy (2001) indicated mortality and morbidity rates from various cancers, circulatory, and respiratory disorders were higher in Windsor than in the rest of the province of Ontario. This work aroused a lot of public sentiments, and several calls were made for further investigation into the “alarming trends” of morbidity and mortality. To respond to the call for an in-depth analysis of the health of Windsorites, we assessed the association between daily ambient air quality and cardiovascular disease hospitalization (Fung et al. 2005). We reported, among other things, that short-term effects of sulfur dioxide were associated significantly with daily cardiac hospital admissions for people ≥65 years of age. The main focus of this article is on respiratory diseases. We used the most recent hospitalization data available from 1995 through 2000 to quantify the association between ambient air pollution and respiratory hospitalization, with temperature, humidity, and change in barometric pressure as covariates. We are especially interested in investigating whether there is an age or sex difference in respiratory admissions. This research will provide policy makers as well as the public with estimates of current risks of respiratory hospitalization as a result of poor ambient air quality.

## Materials and Methods

### Data acquisition.

The study population consisted of all people who were admitted into one of the four hospitals in Windsor with primary diagnoses of respiratory disease [*International Classification of Diseases, 9th Revision* (ICD-9) codes 460–519 (World Health Organization [WHO] 1975)] from 1 April 1995 through 31 December 2000 and were registered with the Ontario Health Insurance Plan (OHIP). Daily hospital admission records for OHIP patients were obtained from the Canadian Institute for Health Information (CIHI) Discharge Abstract Database (CIHI 2002). The data included date of respiratory admission, age, and sex. Our analysis focused on finding the association between air pollution and daily respiratory hospitalizations. It was not able to address events that happened after admission.

The hourly air pollution data from the four fixed monitoring stations in Windsor were obtained from the Ontario Ministry of the Environment (MOE 2000). To capture the effects of exposure, the highest reading for each day was used for the analysis (see Chock et al. 2000). The pollutants were nitrogen dioxide, SO_2_, carbon monoxide, ozone, inhalable particles [particulate matter ≤10 μm in diameter (PM_10_)], coefficient of haze (COH), and total reduced sulfur compounds (TRS). We included COH in our analysis following the recommendation by Goldberg et al. (2001). According to Goldberg et al. (2001), despite the infrequent use of the COH in time-series analyses, it is a reliable measure of the concentration of ambient carbon particles (generally from internal combustion), with only limited contributions from other pollutants, such as sulfates, nitrates, or particle mass. Respirable particles (PM ≤2.5 μm in diameter) data were available only from 1998 through 2001 and were not included in our analysis. Daily weather data including maximum and minimum temperature, humidity, and change in maximum or minimum barometric pressure from the previous day were obtained from the Environment Canada (2002).

### Statistical analysis.

First, we linked together > 2,000 days of records from several databases comprising pollutants, temperature, humidity and pressure, and number of respiratory admissions. Data from CIHI were given to us in a ready-to-use format. Because we used the maximum of daily hourly pollutant values from four stations, there were not many missing values (< 1%). If missing values were sporadic, we replaced the missing values by the mean of nearby points (3 days before and 3 days after). If missing values occurred for a series of days, we substituted the linear trend value for those points using other pollutants and covariates as predictors. In very few cases, if the highest hourly maximum was deemed extreme, it was replaced by the next highest value.

To relate short-term effects of air pollution on the number of respiratory hospitalizations, we used two different statistical techniques: time-series and case crossover methods. Both procedures have been used extensively to analyze this type of data (Burnett et al. 1994; Goldberg et al. 2001; Lee and Schwartz 1999; Lin et al. 2002, 2004; Neas et al. 1999). Detailed formulas are available in the literature.

Since 2002, significant developments in these methodologies have taken place. For time series, the usual smoothing method that has been used for producing residuals with no seasonality was locally weighted regression smoothers (LOESS) within the generalized additive models (GAMs) (Hastie and Tibshirani 1990). It was later discovered (Dominici et al. 2002; Ramsay et al. 2003; Samet et al. 2003) that the default settings of the GAM function in the software package S-Plus (Insightful Corp. 2001) do not assure convergence of its iterative estimation procedure and can provide biased estimates of regression coefficients and standard errors, especially when the concurvity is high. Dominici et al. (2002) reanalyzed the National Morbidity, Mortality, and Air Pollution Study data with the default implementation and found that the estimates were biased upward (i.e., higher than they should be). Since then, either the default option was set to a smaller number, such as 10^−8^ (S-Plus has already done that in their new release), or another smoother called natural splines has been used in the general linear model function.

For case-crossover analysis, Navidi modified his bidirectional design (Navidi 1998) and proposed the semisymmetric bidirectional design (Navidi and Weinhandl 2002). Fung et al. (2003) compared all these methods using simulations, and we used what was recommended in that report—natural splines (ns) in time series and bidirectional case crossover.

For the time-series analysis in this article, daily concentrations of each pollutant and covariates were related to the natural logarithm of hospital admissions, *y*, by the model


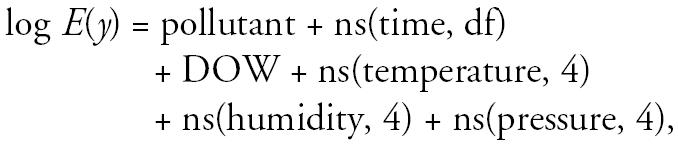


where *E*(*y*) is the mean of *y* and DOW is the day-of-the-week effect, which takes on values 1–7. For each age and sex group, we first found the degrees of freedom (df) for ns(time) such that after fitting the smoothed time effect and DOW, we had a time series of residuals that is as close to white noise as possible, as determined by Bartlett’s test (Priestly 1981). We then extended the model by incorporating the smoothed weather variables. Different combinations of smoothed weather variables (minimum or maximum temperature, humidity, and change in barometric pressure) were examined, and the combination that yielded the lowest Akaike Information Criterion (Akaike 1973) was chosen. Last, we added the air pollutant into the model. Regression models with current-day pollution value (lag 1), average of current day and yesterday (lag 2), and average of current and 2 previous days (lag 3) were examined. Relative risk (RR) was calculated as exp(β̂× IQR), where β̂ is the estimated regression coefficient for pollutant in the above log-linear model and IQR is the interquartile range (75th percentile to 25th percentile) of the pollutant. This implies that the percentage change in the mean number of daily hospitalizations is (RR − 1) × 100% for an increase of IQR unit of pollutant. Ninety-five percent confidence intervals (CIs) of the RRs were obtained under the assumption that the estimated regression coefficients were normally distributed.

The case-crossover design of Maclure (1991) has recently been suggested as an alternative to time-series analysis. This design is essentially a case–control design in which cases serve as their own controls. Risk estimates are based on within subject comparisons of exposures at failure times with exposure at times both before and after failure, using matched case–control methods. This procedure is used to investigate whether a recent exposure has triggered the occurrence of a particular adverse health outcome and is particularly useful for estimating effects that are transient or acute. Because each subject serves as its own control, the case-crossover approach controls for effects of stable subject specific covariates such as sex and race, and for potential time varying confounders such as seasonal effects or personal habits such as smoking. In this study, we used the bidirectional design (Navidi 1998), which can control for different patterns of time trends in exposures and outcomes and gives the least biased estimate compared with the pre- or post-unidirectional design (Fung et al. 2003). We selected an interval of 2 weeks between case and control periods to minimize autocorrelation between case and control exposures and to control for seasonal effects. Conditional logistic regression analysis using the same covariates as time series were performed via the Cox proportional hazards model. Maximum likelihood estimates of the parameters were obtained by choosing the “exact” option in S-Plus. Details of this model can be found in Navidi (1998) or Fung et al. (2003).

## Results

A total of 4,214 overall admissions due to respiratory diseases occurred in the study period. Table 1 gives the summary statistics of daily respiratory admissions for the three age groups (0–14, 15–64, ≥65 years). Overall, there seem to be more male hospitalizations than female in the early years, but the opposite is true for later years. Summary statistics of weather variables and daily high concentrations of all the pollutants are also provided in Table 1. An analysis of the Windsor yearly air pollution data for the period 1990–2000 showed an overall decreasing trend in ambient air pollutants (NO_2_, SO_2_, CO, COH), likely due to regulatory measures implemented by the government in the preceding 10 years (MOE 2000). There was an increasing trend in O_3_ and TRS, whereas PM_10_ did not change much. Based on the air quality index, there were 165 days of poor air quality, 583 days of moderate air quality, and 1,352 days of good air quality during the entire study period.

Table 2 gives the correlation coefficients for the air pollutants and weather variables. Most of the pollutants are positively correlated with each other, except SO_2_ and O_3_ (*r* = −0.02), and TRS and O_3_ (*r* = −0.01). Maximum temperature and minimum humidity were highly correlated with O_3_.

Tables 3 and 4 give the time-series and case-crossover RR estimates by age and sex groups. 95% CIs were also given for the current day (lag 1), lag 2, and lag 3 of the pollutants that were used in the analyses.

The time-series analysis showed elevated effects of NO_2_ on the respiratory admissions of females overall and the 0–14 and 15–64 age groups (Table 3). The results of the case-crossover analysis somewhat concurred with those of the time series. We found NO_2_ lag 2 to be significantly associated with respiratory hospitalization of females 0–14 years age, with an RR of 1.19 (95% CI, 1.002–1.411) (Table 4). Although the effects of NO_2_ on omen in the 15–64 and ≥ 65 age groups were all elevated, none of these were significant. There were no significant associations between NO_2_ and male hospitalization in any of the age groups (Tables 3 and 4).

Time-series results showed a significant current-day effect of SO_2_ on the admission of females 0–14 years of age, with an RR of 1.11(95% CI, 1.011–1.221). The case-crossover method also showed an RR of 1.12, and it is almost significant. Other than this, there were no significant association between SO_2_ and hospitalization for respiratory diseases in females and males using both methods of analysis. However, the effects of SO_2_ on female respiratory admissions were consistently elevated in all age groups.

Although the time-series analysis showed elevated effects of CO on respiratory hospitalization of females, only CO lag 2 was significantly associated with the hospitalization of females 0–14 years of age (RR = 1.07; 95% CI, 1.001–1.139). The case-crossover results showed that CO had both immediate and delayed effects on respiratory admissions for females 0–14 years of age, with RRs of 1.15 (95% CI, 1.006–1.307), 1.19 (95% CI, 1.020–1.379), and 1.22 (95% CI, 1.022–1.459) for lags 1, 2, and 3, respectively. The effects of CO on the respiratory admissions of females in the 15–64 and ≥ 65 age groups were elevated, but none were significant. There were no significant associations between CO and respiratory admissions in any of the male age groups.

We also found no significant association between O_3_ and respiratory admissions on either females or males, although the effects were elevated mostly among the young and elderly age groups in the case-crossover analysis.

The time-series results showed that PM_10_ lag 2 is significantly associated with respiratory hospitalization for males 15–64 years of age, with an RR of 1.18 (95% CI, 1.036–1.332). In the case-crossover analysis, the effects of PM_10_ on respiratory admissions were mostly elevated, but not significant, in all the groups except for males 0–14 years of age.

COH (lag 3) was significantly associated with the admission of all females (RR = 1.07; 95% CI, 1.004–1.135) and for females 15–64 years of age (RR = 1.15; 95% CI, 1.020–1.296) in the time-series analysis. When all the age groups were combined, the case-crossover analysis also showed that COH had an immediate effect on the admission of women for respiratory disease, with an RR of 1.09 (95% CI, 1.037–1.176). COH lags 2 and 3 were also significantly associated with respiratory admissions for females 15–64 years of age, with RRs of 1.20 (95% CI, 1.003–1.426) and 1.29 (95% CI, 1.051–1.582) respectively. None of the effects of COH on the hospitalization of females 0–14 and ≥ 65 years of age for respiratory disease was significant. Furthermore, none of the male groups showed a significant association between COH and respiratory admissions.

Using both methods, we found no significant associations between TRS and respiratory admissions for any group, but the case-crossover results suggested there might be a delayed effect of TRS on the younger age groups.

Taken together, both the time-series and case-crossover analyses show that young (0–14 years) and adult (15–64 years) females were more likely to be admitted for air-pollution–induced respiratory diseases than were males.

## Discussion

Although ambient pollution levels (NO_2_, SO_2_, CO, COH) in Windsor “area of concern” decreased during the study period, we still see existing levels of some pollutants that had significant effects on respiratory hospitalization. Consistent with Lin et al. (2002), we saw some differences in results between time-series and case-crossover analyses. CIs on RR estimates from the bidirectional case-crossover analysis were slightly wider than those from time series, implying lower statistical power for the bidirectional case-crossover design, as documented previously (Bateson and Schwartz 1999; Fung et al. 2003). Because of the sex dimension we introduced into our analysis, together with differences in analytical approaches, control variables, populations studied, exposure variable averaging times, and cut points, comparison of our findings with other studies is not entirely straightforward (e.g., Burnett et al. 1997). Despite the fact that no comparable RRs can be given, our findings are consistent with those of existing studies qualitatively.

Although NO_2_ has been known to increase susceptibility to respiratory infections (Speizer et al. 1980), results of different studies that examined the link between NO_2_ and respiratory outcomes continued to vary. For instance, Atkinson et al. (1999), working in London, reported no significant associations between NO_2_ and respiratory admissions overall or within any of three age groups (0–14, 15–64, and ≥65 years). As part of the Air Pollution and Health: A European Approach (APHEA) project, Spix et al. (1998) reported no significant association between NO_2_ and respiratory admissions for the 15–64 and ≥65 year age groups. In Paris, France (Dab et al. 1996), and in Birmingham, England (Wordley et al. 1997), a lack of associations between NO_2_ and hospital admissions for respiratory diseases was observed. On the other hand, Wong et al. (1999) reported significant associations between NO_2_ and respiratory admissions for 0–4, 5–64 and ≥65 year age groups in Hong Kong. Similarly, in London, England, Ponce de Leon et al. (1996) found a significant association between summer exposure to NO_2_ lag 2 and respiratory admissions for children 0–14 years of age. In the present analysis, we found a significant association between NO_2_ lag 2 and respiratory admissions for females 0–14 years of age, but not for any of the other female or male groups.

The effect of SO_2_ on respiratory hospitalization varies considerably, especially at low levels of exposure. For example, Spix et al. (1998), Sunyer et al. (2003), and Wordley et al. (1997) reported no consistent association between SO_2_ and respiratory admissions. However, studies in Milan, Italy (Vigotti et al. 1996), in Paris, France (Dab et al. 1996), and in London, England (Walters et al. 1994), found SO_2_ levels influenced hospital admissions for all respiratory diseases. Atkinson et al. (1999) reported a strong association between SO_2_ and respiratory admissions among 0- to 14-year-olds. Wong et al. (1999) observed significant short-term effects between SO_2_ and respiratory admissions in the ≥ 65 age group but not among younger age groups. Furthermore, Ponce de Leon et al. (1996) found a positive association between SO_2_ lag 1 (in cool season) with respiratory admissions for adults 15–64 years of age; there was no significant association in either the 0–14 or ≥65 age groups. Bates and Sizto (1987) found an association between SO_2_ (2-day lag) and respiratory admissions in southern Ontario. Consistent with these findings, the time-series analysis in this study showed a significant association between SO_2_ (lag 1) and respiratory admissions for females 0–14 years of age. However, the significance of SO_2_ in all other age groups may be minimal because ambient concentrations of SO_2_ in Ontario have decreased by more than 86% over recent decades (MOE 2000). Nonetheless, there is a need for continuous attention because of the number of people exposed and the existence of high-risk groups.

According to Burnett et al. (1999), because there is a strong correlation between CO and other pollutants regularly used in air pollution studies, it is usually difficult to examine the effects of CO independent of all other pollutants. It is therefore not surprising that the literature on the effects of CO on respiratory illness has also been mixed at best. For instance, Atkinson et al. (1999) found no association between CO and respiratory admissions either overall or by age group. However, in Korea, Cho et al. (2000) after controlling for seasonal and temperature effects, found an association between CO and hospital admissions for respiratory disease with RRs ranging from 1.21 to 3.55, depending on whether the area is rural or urban. In this study, we found that females 0–14 years of age were more likely to be admitted as a result of their exposure to CO in both the time-series and case-crossover models, although only CO lag 2 was significant in the former case. Although the effects of CO on respiratory admissions of women ≥ 65 years of age were elevated for all lags, they were not statistically significant. It is important to note that significant reduction in CO had been achieved in the preceding 10 years in Windsor (mean = 1.0 ppm in 1991 to 0.3 in 2000) because of more stringent regulatory effort in air quality (MOE 2000).

There are contrasting reports on the effect of O_3_ on respiratory admissions. For instance, studies in The Netherlands (Schouten et al. 1996), in London (Atkinson et al. 1999) and in Paris (Dab et al. 1996) found no significant associations between O_3_ and respiratory hospitalization. However, Burnett et al. (1997) reported an association between O_3_ and respiratory admissions in several Canadian cities, using data from 1981 through 1991. In Hong Kong, Wong et al. (1999) reported that O_3_ had a significant effect on respiratory admissions with an RR of 1.022. Ponce de Leon et al. (1996) found an association between O_3_ and daily respiratory admissions for the 15–64 and ≥ 65 age groups but not for the 0–14 age group. Spix et al. (1998) observed a consistent association between O_3_ and respiratory admissions in five European cities, and the effects were stronger in the ≥65 age group. In our analysis, we found elevated risk in the 0–14 and ≥65 age groups; however, none of these RRs was statistically significant.

Several studies have reported positive and significant effects of PM_10_ on respiratory admissions. In Toronto, Canada (Burnett et al. 1999), and in Hong Kong (Wong et al. 1999), PM_10_ has been found to be associated with respiratory admissions. A study by Schwartz (1996) in Spokane, Washington (USA), found PM_10_ to be significantly associated with respiratory hospitalization of women ≥65 years of age. The association between PM_10_ and respiratory admission was demonstrated further by Atkinson et al. (1999), who found significant effects in all age groups (0–14, 15–64, and ≥ 65), although the effect was strongest in the 0–14 age group. In the present study, PM_10_ (lag 2) was significantly associated with respiratory admission of males 15–64 years of age. The elevated effects of PM_10_ found in this study for all female age groups and for adult and elderly males are in line with the notion that PM_10_ influences inflammatory mechanisms in respiratory organs (Hitzfeld et al. 1997).

Compared with other pollutants, the effect of COH on respiratory admissions has not been frequently examined (Goldberg et al. 2001). However, one study found that COH was the strongest predictor of hospitalizations for respiratory diseases among particle-related pollutants examined in both single- and multiple-pollutant regression models (Burnett et al. 1997). Consistent with this later report, we found COH to be significantly related to female respiratory hospitalization, and more so among adult females.

Overall, our results show that there were more elevated effects with female respiratory hospitalization in relation to ambient air pollution compared with males. The reasons for these differences are unclear. However, several authors have suggested possible explanations for existing sex differences observed in respiratory health. According to Redline and Gold (1994), sex differences in respiratory diseases relate to differences in hormonal status, potentially influencing airway inflammation and smooth muscle and vascular functions. Differences may also be related to differences in the rates of lung growth and decline, and the relative changes in airway and parenchymal size, in females and males. For instance, the deposition of pollution particles in the lung has been shown to vary by sex, with greater lung deposition fractions of 1-μm particles in females compared with males (Kim and Hu 1998; Kohlhaufl et al. 1999), leading to a more female susceptibility to respiratory diseases.

Additionally, despite significant social progress, industrial and domestic jobs continue to be different for men and women. In particular, women as a group are poorer than men and may experience different psychosocial stresses. Also, women usually perform the bulk of child care, cooking, dusting, and vacuum cleaning. It is therefore possible that women experience greater exposures to viral infections, nitrogen oxides, household irritants, and aeroallergens (Redline and Gold, 1994). Moreover, some studies have shown that women are more sensitive than men to the effect of smoking, with the effects of smoking on lung function greater in women than in men (e.g., Chen et al. 1999; Prescott et al. 1997; Xu et al. 1994). The increased probability of female hospitalization for respiratory disease probably reflects the increase in smoking among women, relative to men, in the last half-century (Canadian Council for Tobacco Control 2002).

Sex differences in respiratory morbidity, may also reflect differences in the management of morbidity. For instance, Goodman et al. (1994) suggested that increased asthma morbidity in women may relate to their less adequate medical management. The complex social and biologic differences in women and men, underscore the need for more work to aid in our understanding of the bases for a female susceptibility to respiratory diseases.

Limitations of this study are the same as in studies of this kind. They include the adequacy of covariate control and the impact of measurement error in the exposure and outcome variables. However, for most of the risk factors such as the presence of chronic conditions and cigarette smoking, there is no reason to believe that the individual risk factors are correlated with the daily changes in air pollution; hence, they are not likely to be confounders in this study. Furthermore, the limitations of using fixed monitors to represent the entire population in environmental exposure studies have been frequently discussed (Goldberg et al. 2001). Hence, these results must be interpreted with caution. Nevertheless, the findings still have implications for public health policy.

## Conclusion

This study has found associations between ambient air pollution (NO_2_, SO_2_, CO, COH, and PM_10_) and daily hospital admission of respiratory diseases especially among females in the Windsor “area of concern.” The findings are generally consistent with other studies. Even though the risks of respiratory disease due to ambient air pollution in the general population may seem low, it is reasonable to assume that the risks are much higher among susceptible groups, and our findings here support this hypothesis especially for females in the 0–14 age group. Hence, we recommend that in addressing the intense public concern about the health impacts of environmental quality in this “area of concern” must not only involve stricter guidelines (which will be beneficial) but also include environmental risk communication, aimed at improving public perception of risk due to poor air quality. In addition, the events of 11 September 2001 brought renewed concerns about the effects of air pollution in the Windsor area. There have been increasing delays resulting in long lines of trucks at the border crossing points. The idling trucks are spewing toxic pollutants from their archaic exhaust systems into the air. With Windsor located on the downwind side of Detroit, which is a major source of industrial pollutants, the combined effect of these factors is that the improvements that have been suggested here may no longer be possible to attain. We recommend that more frequent studies examining the link between ambient air quality and health effects be conducted to monitor any changes that may be taking place.

## Figures and Tables

**Table 1:** Summary statistics of the daily high concentrations of air pollutants and respiratory admissions, 1 April 1995 through 31 December 2000.

Variable (unit)	Mean ± SD	Minimum	Maximum (AAQC*^a^*)
0–14 years			
Female (*n* = 626)	0.33 ± 0.60	0	4
Male (*n* = 976)	0.52 ± 0.79	0	6
15–64 years			
Female (*n* = 573)	0.30 ± 0.56	0	4
Male (*n* = 310)	0.16 ± 0.41	0	3
≥ 65 years			
Female (*n* = 938)	0.50 ± 0.75	0	5
Male (*n* = 791)	0.42 ± 0.66	0	5
Total (*n* = 4,214)	2.23 ± 1.76	0	14
SO_2_ (ppb)	27.5 ± 16.5	0	129 (100/24 hr)
NO_2_ (ppb)	38.9 ± 12.3	0	117 (100/24 hr)
O_3_ (ppb)	39.3 ± 21.4	1	129 (80/hr)
CO (ppm)	1.3 ± 1.0	0	11.82 (3/hr)
TRS (ppb)	8.1 ± 10.6	0	132 (27/hr)
PM_10_ (μg/m^3^)	50.6 ± 35.5	9	349 (30/24 hr)
COH	0.6 ± 0.4	0	3.6 (1.0/24 hr)
Maximum temperature (°C)	14.2 ± 11.2	−15.8	35.7
Minimum temperature (°C)	5.3 ± 9.8	−21.4	25.6
Maximum humidity	86.1 ± 9.2	50.0	100.0
Minimum humidity	53.4 ± 15.0	17.0	98.0
Maxp	0.00 ± 0.54	−2.36	2.06
Minp	0.00 ± 0.70	−3.42	3.12

Abbreviations: Maxp, change in maximum barometric pressure from the previous day; Minp, change in minimum barometric pressure from the previous day.

a Ambient air quality criteria (MOE 2000).

**Table 2:** Correlation coefficients between air pollutants and weather variables.

	NO_2_	SO_2_	CO	O_3_	COH	PM_10_	TRS	Mint	Minh	Maxt	Maxh	Maxp	Minp
PM_10_													
NO_2_	1.00												
SO_2_	0.22	1.00											
CO	0.38	0.16	1.00										
O_3_	0.26	−0.02	0.10	1.00									
COH	0.49	0.14	0.31	0.23	1.00								
PM_10_	0.33	0.22	0.21	0.33	0.39								
TRS	0.06	0.13	0.11	−0.01	0.15	0.05	1.00						
Mint	−0.22	−0.12	−0.06	−0.45	−0.16	−0.26	−0.10	1.00					
Minh	0.06	−0.06	0.02	0.67	0.21	0.25	0.08	−0.19	1.00				
Maxt	0.15	−0.01	0.08	0.74	0.28	0.34	0.06	0.95	−0.34	1.00			
Maxh	−0.09	−0.08	0.03	−0.20	0.03	−0.09	0.09	−0.02	0.63	−0.07	1.00		
Maxp	−0.06	−0.03	−0.08	−0.04	−0.05	−0.14	−0.02	−0.13	−0.18	−0.14	−0.23	1.00	
Minp	−0.03	−0.01	−0.04	−0.04	−0.05	−0.13	0.04	−0.13	−0.18	−0.15	−0.27	0.67	1.00

Abbreviations: Maxh, maximum humidity; Maxp, change in maximum barometric pressure from the previous day; Maxt, maximum temperature; Minh, minimum humidity; Minp, change in minimum barometric pressure from the previous day; Mint, minimum temperature.

**Table 3:** RRs (95% CIs) for single-pollutant models using time-series method for an increase in IQR.*^a^*

	All age groups		0–14 years		15–64 years		≥65 years	
Pollutants (IQR)	Female	Male	Female	Male	Female	Male	Female	Male
NO_2_ (16 ppb)								
Lag 1	1.035 (0.971–1.104)	0.944 (0.886–1.006)	1.114 (0.994–1.248)	0.955 (0.866–1.054)	1.013 (0.893–1.150)	0.942 (0.790–1.122)	1.020 (0.930–1.1198)	0.9196 (0.832–1.016)
Lag 2	1.027 (0.967–1.094)	0.958 (0.900–1.021)	1.107 (0.990–1.238)	0.918 (0.833–1.012)	1.044 (0.918–1.187)	0.992 (0.833–1.182)	0.987 (0.881–1.106)	0.9620 (0.854–1.084)
Lag 3	1.036 (0.970–1.107)	0.970 (0.909–1.036)	1.108 (0.987–1.245)	0.927 (0.838–1.025)	1.121 (0.978–1.285)	1.012 (0.841–1.216)	0.962 (0.847–1.093)	0.9773 (0.854–1.118)
SO_2_ (19.25 ppb)								
Lag 1	1.041 (0.987–1.098)	0.953 (0.900–1.009)	1.111 (1.011–1.221)*	0.952 (0.874–1.037)	1.031 (0.930–1.144)	0.971 (0.845–1.115)	1.030 (0.951–1.115)	0.9409 (0.860–1.029)
Lag 2	1.041 (0.979–1.107)	0.984 (0.925–1.048)	1.090 (0.977–1.216)	0.981 (0.892–1.078)	1.068 (0.950–1.202)	1.046 (0.898–1.218)	1.030 (0.927–1.145)	0.9490 (0.845–1.066)
Lag 3	1.046 (0.982–1.114)	0.987 (0.925–1.053)	1.066 (0.952–1.194)	0.995 (0.904–1.096)	1.054 (0.931–1.192)	0.985 (0.837–1.159)	1.074 (0.949–1.215)	0.9561 (0.834–1.096)
CO (1.17 ppm)								
Lag 1	1.049 (0.993–1.108)	0.989 (0.932–1.049)	1.077 (0.979–1.184)	1.034 (0.949–1.126)	1.072 (0.962–1.195)	0.994 (0.854–1.157)	1.029 (0.947–1.118)	0.9010 (0.817–0.994)
Lag 2	1.032 (0.993–1.188)	0.986 (0.946–1.029)	1.068 (1.001–1.139)*	0.996 (0.933–1.062)	1.025 (0.944–1.112)	0.988 (0.884–1.104)	1.030 (0.928–1.144)	0.9041 (0.803–1.019)
Lag 3	1.051 (0.993–1.112)	0.987 (0.929–1.048)	1.100 (0.997–1.213)	0.968 (0.881–1.064)	1.081 (0.963–1.213)	0.951 (0.806–1.121)	1.013 (0.899–1.142)	0.9632 (0.845–1.098)
O_3_ (29 ppb)								
Lag 1	0.947 (0.819–1.096)	1.039 (0.923–1.170)	1.048 (0.830–1.322)	0.944 (0.745–1.196)	0.817 (0.621–1.075)	0.959 (0.661–1.393)	0.945 (0.777–1.150)	1.0961 (0.920–1.306)
Lag 2	1.006 (0.852–1.188)	1.063 (0.917–1.232)	1.084 (0.829–1.433)	0.955 (0.731–1.246)	0.759 (0.549–1.048)	1.268 (0.832–1.932)	1.008 (0.807–1.259)	1.0624 (0.852–1.325)
Lag 3	1.043 (0.873–1.246)	1.057 (0.891–1.254)	1.092 (0.796–1.497)	1.001 (0.755–1.328)	0.893 (0.633–1.261)	1.346 (0.851–2.128)	0.963 (0.763–1.215)	0.9767 (0.757–1.261)
PM_10_ (31 μg/m^3^)								
Lag 1	0.996 (0.950–1.044)	1.008 (0.965–1.054)	1.023 (0.948–1.104)	0.980 (0.912–1.053)	1.047 (0.962–1.140)	1.096 (0.982–1.222)	0.967 (0.900–1.040)	1.0033 (0.934–1.078)
Lag 2	1.015 (0.963–1.069)	1.036 (0.986–1.089)	1.035 (0.948–1.130)	1.001 (0.925–1.083)	1.049 (0.946–1.163)	1.175 (1.036–1.332)*	0.993 (0.913–1.079)	1.0298 (0.941–1.127)
Lag 3	1.022 (0.968–1.078)	1.027 (0.974–1.083)	1.047 (0.956–1.147)	0.980 (0.901–1.065)	1.030 (0.922–1.150)	1.080 (0.938–1.243)	0.998 (0.910–1.094)	1.0768 (0.972–1.193)
COH (0.5)								
Lag 1	1.051 (0.994–1.113)	0.977 (0.922–1.036)	1.085 (0.986–1.195)	1.004 (0.923–1.093)	1.103 (0.994–1.223)	0.955 (0.820–1.113)	0.996 (0.912–1.088)	0.9381 (0.852–1.033)
Lag 2	1.032 (0.982–1.086)	0.991 (0.942–1.043)	1.066 (0.979–1.161)	0.980 (0.907–1.058)	1.056 (0.958–1.164)	0.996 (0.871–1.141)	0.989 (0.884–1.107)	0.9841 (0.876–1.106)
Lag 3	1.067 (1.004–1.135)*	1.001 (0.940–1.066)	1.094 (0.985–1.216)	0.972 (0.884–1.070)	1.150 (1.020–1.296)*	0.948 (0.799–1.126)	0.998 (0.875–1.137)	1.0609 (0.928–1.213)
TRS (8 ppb)								
Lag 1	0.990 (0.939–1.038)	0.997 (0.961–1.035)	0.957 (0.887–1.031)	0.993 (0.938–1.052)	1.013 (0.942–1.090)	0.981 (0.896–1.074)	0.997 (0.945–1.051)	1.0126 (0.958–1.070)
Lag 2	0.987 (0.939–1.038)	0.999 (0.950–1.051)	1.002 (0.913–1.100)	0.982 (0.908–1.063)	1.023 (0.926–1.130)	1.015 (0.904–1.140)	0.961 (0.892–1.034)	1.0089 (0.9341–1.090)
Lag 3	0.976 (0.924–1.032)	1.003 (0.949–1.060)	1.063 (0.965–1.171)	0.990 (0.909–1.079)	0.980 (0.874–1.100)	0.988 (0.866–1.128)	0.925 (0.845–1.011)	1.0227 (0.934–1.120)

a Adjusted for temperature, humidity, and change in barometric pressure.

* Statistically significant at *p* < 0.05.

**Table 4:** RRs (95% CIs) for single-pollutant models using case-crossover method for an increase in IQR.*^a^*

	All age groups		0–14 years		15–64 years		≥65 years	
Pollutants (IQR)	Female	Male	Female	Male	Female	Male	Female	Male
NO_2_ (16 ppb)								
Lag 1	1.078 (0.995–1.168)	0.957 (0.883–1.036)	1.145 (0.996–1.317)	0.981 (0.873–1.103)	1.004 (0.870–1.159)	0.988 (0.806–1.210)	1.081 (0.964–1.212)	0.915 (0.810–1.034)
Lag 2	1.100 (0.998–1.213)	0.960 (0.873–1.055)	1.189 (1.002–1.411)*	0.933 (0.810–1.074)	1.055 (0.883–1.260)	1.004 (0.789–1.277)	1.063 (0.925–1.222)	0.959 (0.832–1.105)
Lag 3	1.085 (0.972–1.210)	0.951 (0.854–1.057)	1.178 (0.973–1.427)	0.910 (0.777–1.066)	1.114 (0.915–1.356)	0.972 (0.744–1.268)	1.001 (0.856–1.172)	0.973 (0.829–1.142)
SO_2_ (19.25 ppb)								
Lag 1	1.047 (0.978–1.122)	0.939 (0.874–1.009)	1.119 (0.995–1.259)	0.923 (0.831–1.025)	1.002 (0.879–1.141)	0.944 (0.798–1.116)	1.020 (0.924–1.126)	0.968 (0.867–1.082)
Lag 2	1.062 (0.969–1.164)	1.003 (0.914–1.101)	1.126 (0.957–1.325)	0.984 (0.859–1.128)	1.057 (0.893–1.252)	1.071 (0.859–1.334)	1.011 (0.888–1.152)	0.994 (0.861–1.147)
Lag 3	1.073 (0.963–1.195)	0.989 (0.886–1.103)	1.100 (0.907–1.335)	0.961 (0.819–1.126)	1.055 (0.864–1.289)	1.022 (0.785–1.330)	1.044 (0.896–1.216)	1.008 (0.852–1.192)
CO (1.17 ppm)								
Lag 1	1.037 (0.968–1.111)	0.950 (0.884–1.020)	1.147 (1.006–1.307)*	1.003 (0.904–1.113)	1.005 (0.884–1.141)	1.036 (0.870–1.233)	1.014 (0.922–1.116)	0.867 (0.775–0.970)
Lag 2	1.063 (0.976–1.158)	0.945 (0.862–1.036)	1.186 (1.020–1.379)*	0.997 (0.871–1.141)	1.007 (0.859–1.181)	1.033 (0.821–1.299)	1.024 (0.907–1.156)	0.865 (0.752–0.994)
Lag 3	1.087 (0.982–1.203)	0.965 (0.866–1.075)	1.221 (1.022–1.459)*	0.970 (0.824–1.141)	1.032 (0.858–1.240)	0.991 (0.760–1.293)	1.035 (0.893–1.200)	0.946 (0.807–1.109)
O_3_ (29 ppb)								
Lag 1	1.013 (0.766–1.339)	1.064 (0.930–1.217)	1.046 (0.800–1.367)	1.070 (0.854–1.340)	0.937 (0.723–1.214)	0.899 (0.630–1.282)	1.122 (0.919–1.369)	1.095 (0.896–1.339)
Lag 2	1.066 (0.778–1.462)	1.037 (0.889–1.211)	1.084 (0.797–1.474)	1.024 (0.797–1.316)	0.838 (0.625–1.123)	0.974 (0.651–1.457)	1.147 (0.912–1.444)	1.039 (0.826–1.308)
Lag 3	1.007 (0.712–1.424)	1.015 (0.855–1.207)	1.013 (0.721–1.425)	1.032 (0.786–1.355)	0.877 (0.639–1.203)	0.972 (0.625–1.513)	1.161 (0.901–1.496)	0.987 (0.765–1.273)
PM_10_ (31 μg/m^3^)								
Lag 1	1.034 (0.974–1.098)	0.997 (0.942–1.056)	1.040 (0.944–1.146)	0.965 (0.887–1.050)	1.038 (0.937–1.151)	1.055 (0.926–1.203)	1.027 (0.936–1.125)	0.999 (0.912–1.094)
Lag 2	1.045 (0.972–1.124)	1.022 (0.953–1.097)	1.032 (0.916–1.162)	0.948 (0.857–1.048)	1.051 (0.920–1.200)	1.136 (0.964–1.339)	1.051 (0.943–1.171)	1.059 (0.942–1.191)
Lag 3	1.054 (0.970–1.145)	1.008 (0.930–1.092)	1.052 (0.919–1.204)	0.914 (0.815–1.025)	1.020 (0.872–1.194)	1.026 (0.852–1.236)	1.073 (0.949–1.214)	1.125 (0.985–1.284)
COH (0.5)								
Lag 1	1.092 (1.037–1.176)*	0.974 (0.906–1.048)	1.101 (0.971–1.245)	1.025 (0.925–1.134)	1.135 (0.997–1.292)	1.013 (0.845–1.214)	1.058 (0.946–1.184)	0.898 (0.799–1.008)
Lag 2	1.097 (0.998–1.206)	1.001 (0.913–1.098)	1.119 (0.953–1.314)	1.004 (0.880–1.144)	1.196 (1.003–1.426)*	1.040 (0.823–1.315)	1.029 (0.897–1.181)	0.966 (0.837–1.115)
Lag 3	1.104 (0.989–1.232)	1.020 (0.915–1.136)	1.086 (0.903–1.307)	0.995 (0.853–1.160)	1.289 (1.051–1.582)*	0.968 (0.740–1.267)	1.016 (0.865–1.193)	1.048 (0.886–1.241)
TRS (8 ppb)								
Lag 1	1.007 (0.961–1.054)	0.990 (0.945–1.037)	0.982 (0.899–1.072)	0.991 (0.923–1.063)	0.985 (0.903–1.076)	0.994 (0.895–1.103)	1.030 (0.965–1.098)	0.990 (0.925–1.061)
Lag 2	1.000 (0.940–1.064)	1.009 (0.948–1.075)	1.056 (0.941–1.184)	1.015 (0.921–1.118)	0.960 (0.858–1.074)	1.035 (0.907–1.181)	0.987 (0.903–1.078)	0.992 (0.902–1.092)
Lag 3	1.005 (0.935–1.081)	1.018 (0.944–1.098)	1.144 (0.999–1.310)	1.015 (0.933–1.185)	0.932 (0.813–1.069)	1.016 (0.867–1.192)	0.967 (0.872–1.073)	0.991 (0.886–1.110)

a Adjusted for temperature, humidity, and change in barometric pressure.

* Statistically significant at *p* < 0.05.
